# Research on the Parasitic Inductance of the Bonding Wires in IGBT Modules Based on Their Morphology and Layout

**DOI:** 10.3390/mi17010026

**Published:** 2025-12-25

**Authors:** Junwei Cao, Sheng Wu, Yanhui Wang, Chongyang Xu, Xiaotong Wang, Weibin Jiang, Yingchun Wang, Yuan Wang

**Affiliations:** 1 Yantai Research Institute, Harbin Engineering University, Yantai 370611, China; cccjjjwww1999@163.com (J.C.); wusheng@hrbeu.edu.cn (S.W.); wangyh1987@hrbeu.edu.cn (Y.W.); xcy110@126.com (C.X.); wxthrbeu@hrbeu.edu.cn (X.W.); 2 Yantai Taixin Electronics Technology Co., Ltd., Yantai 370676, China; jiangweibin@yttaixin.com (W.J.); wangyingchun@yttaixin.com (Y.W.); 3 Science and Technology Service Industry Department, Yantai Science and Technology Innovation Promotion Center, Yantai 264003, China

**Keywords:** IGBT modules, bond wires, parasitic inductance, morphology, layout

## Abstract

As a typical electronic switching device, IGBT is widely used in various fields. Reducing the parasitic inductance parameters of IGBT is of great significance for improving the performance of power devices, enhancing system stability and reliability. To study the parasitic inductance of the internal bonding wire connection structure during the operation of IGBT modules, this paper considers the morphological modeling of bonding wires, bonding parameters, and the layout of bonding wire arrays, and proposes a new analysis model. Through mathematical calculation of the analysis model and Ansys Q3D simulation, the bonding wires with different geometric dimensions and layouts were studied and good correlation were obtained. This will reduce the impact of bonding parameters on the total parasitic inductance of the bonding wire during the pre-layout stage.

## 1. Introduction

Insulated Gate Bipolar Transistor (IGBT) combines the excellent characteristics of MOSFET (Metal-Oxide-Semiconductor Field-Effect Transistor) and bipolar transistors, featuring high voltage capability, simple drive, low on-state power consumption, and low resistance. IGBT is typically used as an electronic switching device. Depending on its operating frequency and output capacity, it is widely applied in fields such as motors, electrical appliances, switching power supplies, and electric vehicles [1,2,3].

The bonding wire interconnection method is important for IGBT as well as semiconductor packaging processes. Through fine metal wires (such as gold wires, copper wires or aluminum wires), the pads of the chip are connected to the packaging substrate or lead frame, achieving electrical and mechanical connections [4,5,6,7]. Its advantages include low manufacturing costs, flexible manufacturing processes, high reliability and a mature infrastructure. At higher frequencies, metal bonding wires can generate parasitic inductance, and at the same time, they will cause voltage spikes during the switching process, increase switching losses, electromagnetic interference (EMI), and dynamic current sharing during parallel operation, reducing the parasitic inductance parameters of the packaging is of great significance for improving the performance of power devices, enhancing system stability and reliability [8,9]. Extracting the parasitic inductance of the bonding wires can provide significant assistance in studying the bonding wire fatigue model, failure modes, and electro-thermal performance of IGBT modules [10,11,12].

It is particularly important to note that the long-term reliability of IGBT modules is essentially a challenge involving multiple physical fields, including intricate coupling relationships. Switching losses caused by parasitic inductance generate heat, which over time may alter the resistivity and geometry of the connection lines, ultimately affecting reliability. Although comprehensive reliability co-design requires such coupling analysis, this preliminary study focuses on establishing an accurate and fundamental electromagnetic model. The main objective here is to provide an accurate and efficient method for predicting the parasitic inductance of the bonding wires solely based on their shape and layout. This electromagnetic model is a crucial first step and is a key input parameter for any subsequent high-precision electro-thermal or thermal-mechanical reliability analysis.

In previous studies, many research methods for the parasitic inductance of bonding wires have been proposed. At the very beginning, the calculation formula for wire inductance proposed by Grover [13] was used. In the subsequent research process [14], the bonding wire was often treated as a long straight wire for numerical calculation of parasitic inductance. However, a straight wire cannot represent the actual shape of the bonding wire, so this calculation method inevitably leads to incorrect results. To reduce errors, in [15], the bonding wires were segmented into a series of straight transmission segments for calculation. Furthermore, N. Hassaine et al. [16,17] regarded the shape of the bonding wire as a semicircles/half-circular loops for the calculation of inductance.

The Method of Moments (MoM) was employed to extract the self-inductance and mutual inductance of the bonding wires [18,19]. F. Alimenti et al. [20] proposed the finite-difference time-domain (FDTD) method to study the interconnections of bonding wires from the perspectives of modeling and electrical characterization and considered both single-wire and double-wire electrical models and evaluated the accuracy and applicability of the quasi-static model.

I. Ndip et al. [21] used Gaussian distribution functions to conduct mathematical modeling of the bonding wires. The shape, bonding parameters and materials of the bonding wires have been studied. The maximum deviation between the calculation results of the model and the Ansys Q3D simulation results is approximately 1%, verifying the accuracy of the proposed model.

Comparison of bond wire morphology and calculation method in references is shown is Table 1.

To extract the parasitic inductance of bonding wires quickly and accurately, scholars have proposed many extraction methods based on machine learning, deep learning and neural networks. By training the model, the self-inductance and mutual inductance of the bonding wires can be accurately extracted solely based on their geometric dimensions. This method can save a significant amount of time for analyzing the inductance of bonding wires and designing the overall bonding interconnection [22,23,24,25].

To ensure the application in high-frequency switching, a comprehensive solution for the optimized design of the shape and layout of bonding wires is required [26]. In previous studies, only the single-wire and parallel double-wire electrical structures of bonding wires were studied. In this paper, based on the shape of the bonding wire, the bonding material, and various factors (such as the maximum height $H_{max}$ of the bonding wire, the bonding position spacing dbp, the radius rbw, the thickness of the metallized layer attached to the bonding wire $t_{met}$, the *Z*-axis spacing $p_{z}$, the *X*-axis spacing $p_{x}$, and the deflection Angle *θ*), we accurately and efficiently calculate the parasitic self-inductance and parasitic mutual inductance of the bonding wire. To verify the model, the inductance of interconnected bond wire with different geometric dimensions was analyzed using the model we proposed. Compared with the results of Ansys Q3D, the maximum difference was approximately 1%.

Compared to spending a lot of time starting from modeling the bonding wires, then importing them into Ansys Q3D to set up the program and finally conducting the simulation, simply inputting the relevant parameters in the analysis model can already yield the calculation results. This undoubtedly saves a lot of time and provides a relatively accurate calculation result, preparing for the next step of work [27,28,29].

This work falls within the stage of theoretical model and simulation verification. The contribution lies in the innovation of the model itself and the revelation of its good electrical analysis performance. It should be noted that the manufacturability of complex profile bond lines has been confirmed by previous research [16,17,21,30], which provides a practical foundation for the theoretical exploration in this work.

The structure of the remaining part of this article is arranged as follows: Section 2 briefly introduces the morphological model of the bonding wire, deduces the analytical formula for calculating the parasitic inductance, and verifies the analytical model in the morphology of the bonding wire in Section 3. Section 4 discusses the application of the argumentative model in the layout of bonding wires and presents the measurement results.

## 2. Modeling of Bonding Wire Morphology and Calculation Formula of Parasitic Inductance

In most studies, the modeling of bonding wires uses very simple shapes, such as semicircles/half-circular loops [16,17]. These shapes cannot represent the true shape of the bonding wires because they only involve one parameter, the radius of the circle, to represent the geometry of the wire.

In this section, the Gaussian distribution function is used to mathematically model the bonding wire, and then integrate the parasitic self-inductance and mutual inductance of the bonding wire using the magnetic flux, respectively. This lays a mathematical foundation for the research in this paper.

### 2.1. The Morphological Model of the Bonding Wire

**Figure 1:**
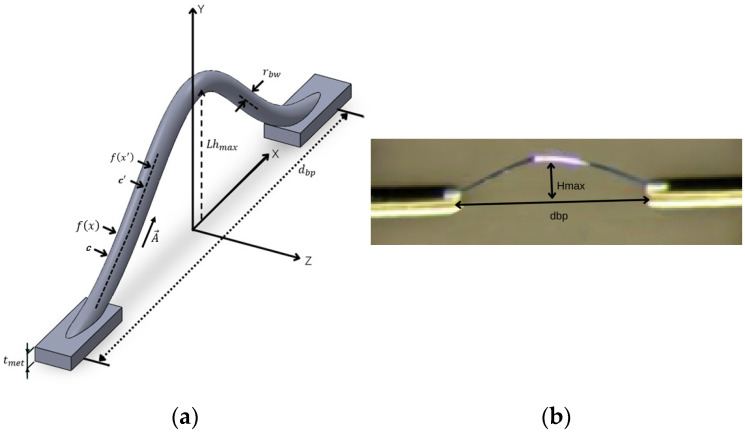
(**a**) The morphological model of the bonding wire; (**b**) Optical photos of the sample with approximately Gaussian-shaped bonding lines prepared by the wedge–wedge bonding process.

As shown in Figure 1a, the Gaussian distribution function is used to mathematically model the bonding wire. The Gaussian model proposed in this study relies on the mature wedge–wedge bonding process for its physical implementation. To demonstrate that it is not merely a theoretical concept, this paper fabricated a bonding line sample with an approximately Gaussian profile based on the parameters of this process (Figure 1b). The successful preparation of this sample indicates that the target geometry can be achieved in principle using standard equipment, preliminarily supporting the feasibility of the model concept. It should be noted that the focus of this study is on the electrical characteristic simulation and comparative analysis of the new model. Figure 1b is intended to qualitatively demonstrate the feasibility of the shape, rather than quantitatively characterizing the manufacturing process tolerance, repeatability, or variability. The latter falls within the scope of process optimization and is an important step in advancing this model to practical application. The figure indicates that the shape of our model is very similar to the shape of the fabricated bonding wire.

The analytical models between the shape ($f\left(x\right)$) and length ($l_{bw}$) of the bonding wire and the bonding parameters are derived using the Gaussian function, as shown in (1) and (2), respectively.

(1) $$f\left(x\right)=\left(H_{max}\right)e^{-\frac{4}{{\left(d_{bp}\right)}^{2}}ln\left(\frac{H_{max}}{t_{met}}\right)x^{2}},$$

and

(2) $$l_{bw}=\int_{u}^{v}\left(\sqrt{1+{\left((-2bx)(ae^{-bx^{2}})\right)}^{2}}\right)dx,$$

where $a={Lh}_{max}$ and $b=-\frac{4}{{\left(d_{bp}\right)}^{2}}ln\left(\frac{H_{max}}{t_{met}}\right)$. *u* and *v* are, respectively, the starting and ending bonding positions of the bonding wire.

In the next section, we will apply the proposed bond wire shape and length model *c’* to derive the analytical model for calculating the interconnection inductance of a single bond wire and a coupled bond wire.

### 2.2. Calculation Formula for Parasitic Self-Induction of Bonding Wires

In this paper, the parasitic self-inductance and mutual inductance of bonding wires are calculated by integrating the magnetic flux. Under low-frequency conditions, when the skin depth (*δ*) is greater than the diameter of the bonding wire, the parasitic self-induction $L_{tps}$ of the bonding wire. It is composed of the external self-inductance $L_{{ps}_{ext}}$ and the low-frequency self-inductance of the wire, as shown in (3).

(3) $$L_{tps\_lf}=L_{{ps}_{ext}}+\frac{\mu_{0}}{8\pi }l_{bw},$$

Under high-frequency conditions, the skin effect needs to be taken into account. when the skin depth (*δ*) is smaller than the diameter, $L_{tps\_hf}$ is composed of $L_{{ps}_{ext}}$ and the high- frequency self-inductance $L_{si\_hf}$. $L_{si\_hf}$ is derived from the high-frequency internal impedance $Z_{i_{hf}}$, as given in (4).

(4) $$L_{si\_hf}=\frac{R_{s}}{4\pi^{2}r_{bw}f}l_{bw},$$

where $R_{s}$ = 1/σδ = $\sqrt{\pi \mu_{0}f/\sigma }$ is the surface resistance of the wire.

Therefore, the parasitic self-induction of the bonding wire is calculated as (5).

(5) $$L_{tps\_hf}=L_{{ps}_{ext}}+\frac{R_{s}}{4\pi^{2}r_{bw}f}l_{bw},$$

Typically, the radius of the bonding wires for power packaging is less than 250 μm. When the switching frequency does not exceed 120 kHz, the skin effect can be disregarded.

**Figure 2:**
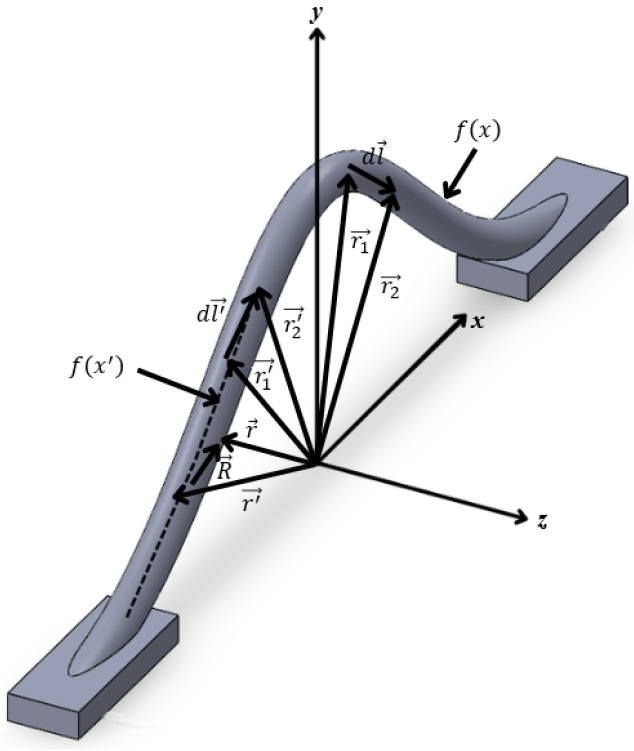
Illustrating the calculation of the partial self-inductance of a bond wire.

As shown in Figure 1a and Figure 2, the external partial self-inductance $L_{{ps}_{ext}}$ of the wire is given as

(6) $$L_{{ps}_{ext}}=\frac{1}{I}\int_{c}\vec{A}\;\cdot \;d\vec{l},$$

where c is the contour along the outer edge of the bond wire, $\vec{A}$ is the magnetic vector potential along c, produced by I, and $d\vec{l}$ is a vector differential length along c. $\vec{A}$ is defined as

(7) $$\vec{A}=\frac{\mu_{0}}{4\pi }\int_{c^{\prime }}\frac{1}{R}\;\cdot \;d\vec{l},$$

where R is the distance between the point where $\vec{A}$ is determined and the differential current segment, $c^{\prime }$ is the contour along the filament current at the center of the wire, and $d\vec{l^{\prime }}$ is a vector differential length along $c^{\prime }$.

Substituting (7) into (6), (8) can be obtained for calculating the $L_{{ps}_{ext}}$.

(8) $$L_{{ps}_{ext}}=\frac{\mu_{0}}{4\pi }\int_{c}\int_{c^{\prime }}\frac{d\vec{l}\cdot d\vec{l^{\prime }}}{R},$$

R, $d\vec{l}$ and $d\vec{l^{\prime }}$ in this case is given as

(9) $$R=|\vec{R}|=|\vec{r}-\vec{r^{\prime }}|,$$

(10) $$\vec{r}=x\vec{a_{x}}+f\left(x\right)\vec{a_{y}}+r_{bw}\vec{a_{z}},$$

(11) $$\vec{r^{\prime }}=x^{\prime }\vec{a_{x}}+f\left(x^{\prime }\right)\vec{a_{y}},$$

(12) $$d\vec{l}=\frac{d(\vec{r})}{dx}dx=\left(\vec{a_{x}}-2abxe^{-bx^{2}}\vec{a_{y}}\right)dx,$$

(13) $$d\vec{l^{\prime }}=\frac{d(\vec{r^{\prime }})}{dx}dx=\left(\vec{a_{x}}-2abx^{\prime }e^{-b{x^{\prime }}^{2}}\vec{a_{y}}\right)dx^{\prime },$$

Substituting (9)–(13) into (8), the (14) for the external self-inductance $L_{{ps}_{ext}}$ of the bonding wire can be obtained.

(14) $$L_{{ps}_{ext}}=\frac{\mu_{0}}{4\pi }\int_{c}\int_{c^{\prime }}\frac{\left(1+{\left(2b(H_{max})\right)}^{2}x^{\prime }xe^{-b(x^{2}+{x^{\prime }}^{2})}\right)}{\sqrt{{\left(x-x^{\prime }\right)}^{2}+{\left(\left(H_{max}\right)e^{-bx^{2}}-\left(H_{max}\right)e^{-b{x^{\prime }}^{2}}\right)}^{2}+{\left(r_{bw}\right)}^{2}}},$$

### 2.3. The Calculation Formula for the Coupling Mutual Inductance of Bonding Wires

**Figure 3:**
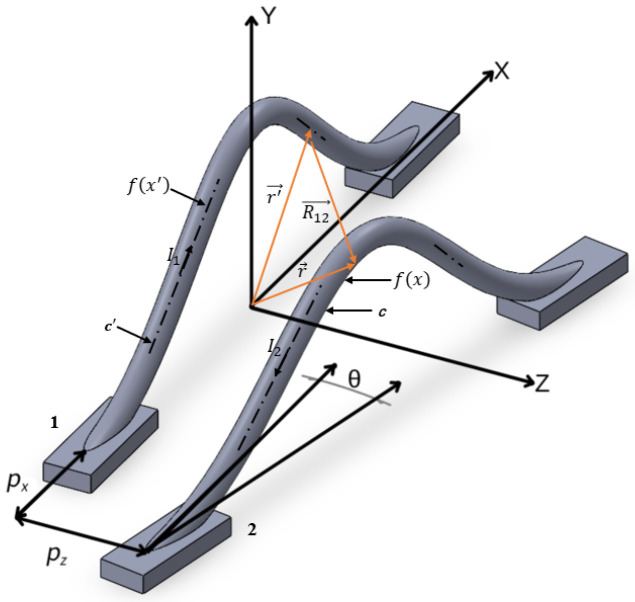
The non-parallel structure model of wire bonding with $p_{z}$, $p_{x}$ and *θ*.

As shown in Figure 3, the $r_{bw}$, $H_{max}$ and $d_{bp}$ of the two bonding wires is the same. The difference lies in that there not only exists the *Z*-axis spacing $p_{z}$, but also the *X*-axis spacing $p_{x}$ and the deflection angle *θ* in space.

When the two bonding wires within the space are parallel, that is, only $p_{z}$ exists and neither $p_{x}$ nor *θ* is present. Here, (8) is used to derive the coupling mutual inductance $L_{pm}$.

At this point, $d\vec{l}$ remains unchanged, while $d\vec{l^{\prime }}$ takes on the corresponding $\vec{r^{\prime }}$ as shown in (15). With all other variables remaining constant, substituting (9), (10), (12) and (15) into (8), the final calculation result is as shown in (16).

(15) $$d\vec{l^{\prime }}=\frac{d(\vec{r^{\prime }})}{dx}dx=\frac{d(x^{\prime }\vec{a_{x}}+f\left(x^{\prime }\right)\vec{a_{y}}+(p_{z}+r_{bw})\vec{a_{z}})}{dx}dx,$$

(16) $$L_{pm}=\frac{\mu_{0}}{4\pi }\int_{c}\int_{c^{\prime }}\frac{\left(1+{\left(2b\left({Lh}_{max}\right)\right)}^{2}x^{\prime }xe^{-b\left(x^{2}+{x^{\prime }}^{2}\right)}\right)}{\sqrt{{\left(x-x^{\prime }\right)}^{2}+{\left(\left(H_{max}\right)e^{-bx^{2}}-\left(H_{max}\right)e^{-b{x^{\prime }}^{2}}\right)}^{2}+{\left(p_{z}+r_{bw}\right)}^{2}}},$$

As shown in Figure 3, there are $p_{z}$, $p_{x}$ and *θ* components in the two bonding wires within the space. The second bonding wire is expressed by parameters as shown in (17).

(17) $$\left\{\begin{array}{c} x^{\prime }=x\\ y^{\prime }=ae^{-b{\left(\frac{x+p_{x}}{cos\theta }\right)}^{2}}\\ z^{\prime }=\left(x+p_{x}\right)tan\theta +p_{z}+\frac{d_{bp}}{2}tan\theta \end{array}\right.,$$

Substituting (17) into (8) to obtain (18) for the $L_{pm}$ between the bonding wires.

(18) $$L_{pm}=\frac{\mu_{0}}{4\pi }\int_{c}\int_{c^{\prime }}\frac{\left(1+4a_{1}b_{1}\frac{a_{2}b_{2}}{cos\theta }x^{\prime }xe^{-b_{1}x^{2}+b_{2}{\left(\frac{x^{\prime }}{cos\theta }\right)}^{2}}\right)}{\sqrt{{\left(x-x^{\prime }\right)}^{2}+{\left(a_{1}e^{-b_{1}x^{2}}-a_{2}e^{-b_{2}{\left(\frac{x^{\prime }}{cos\theta }\right)}^{2}}\right)}^{2}+{\left(x^{\prime }tan\theta +p_{z}+\frac{d_{bp}}{2}tan\theta \right)}^{2}}},$$

## 3. The Influence of the Morphological Model of the Bonding Wire on the Parasitic Inductance

In the previous section, a mathematical model for calculating the parasitic inductance of the bonding wire was derived. In this section, the parameters $r_{bw}$, $H_{max}$, $d_{bp}$, $p_{x}$, and $p_{z}$ of the bonding wire will be considered and verified through ANSYS (Ansys Electronics Suite 2023 R1) Q3D simulation and mathematical calculations.

The simulation conditions in ANSYS Q3D are as follows: the material is aluminum, with no boundary, and the mesh is adaptive. Set one end of each bonding wire as the signal net and the other one as the source. When calculating the total parasitic inductance of the bonding wire array, select JoinParalleMatrix in the Reduce Matrix option, and then select all the bonding wires. In industrial applications, power modules based on IGBT typically operate in the range of 1 kHz to 50 kHz, with 10 to 20 kHz being the typical switching frequency range for medium-high power systems (such as motor drivers, inverters). Thus, in this paper, 10 kHz is chosen as the simulation frequency. In the Solve Setup, we mainly check AC Resistance/Lanductance, and set the Maximum Number of Passes to 20, the Minimum Number of Passes to 1, the Minimum Converged Passes to 2, the Percent Error to 0.1, and the Percent Refinement Per Pass to 30.

### 3.1. Verification of Parasitic Self-Induction of Bonding Wires

In the previous section, the analytical model for calculating the parasitic inductance of a single bonding wire was presented. In this subsection, the influences of $r_{bw}$, $H_{max}$ and $d_{bp}$ on the parasitic self-inductance $L_{tps\_lf}$ under low-frequency conditions are discussed, respectively.

**Figure 4:**
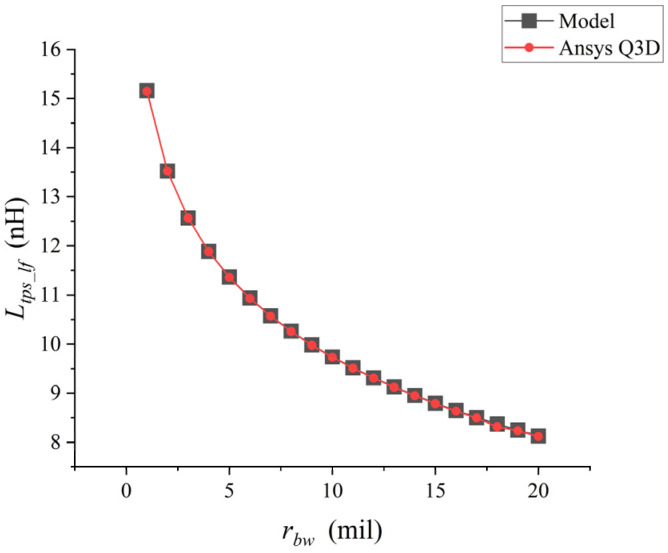
Point plot of the relationship between $r_{bw}$ and $L_{tps\_lf}$.

When the parameters $H_{max}$ and $d_{bp}$ remain unchanged and only $r_{bw}$ is changed, the analytical model is used for calculation in matlab and the simulation is carried out in Ansys Q3D, respectively. The comparison results are shown in Figure 4.

When the diameter of a single bonding wire keeps increasing, $L_{tps\_lf}$ keeps decreasing in Figure 3. The low-frequency self-inductance value of the bonding wire calculated by the analytical model was compared with the inductance value extracted by Ansys Q3D. There was an excellent correlation between the inductance values extracted by the two methods, with a maximum difference of approximately 1%.

**Figure 5:**
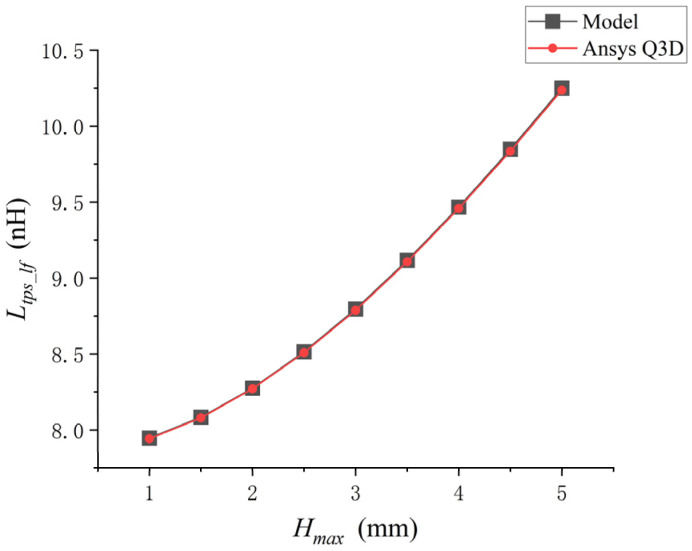
Point plot of the relationship between $H_{max}$ and $L_{tps\_lf}$.

When $r_{bw}$ and $d_{bp}$ remain unchanged and only $H_{max}$ is changed, the analytical model is used for calculation in matlab and the simulation is carried out in Ansys Q3D, respectively. The comparison results are shown in Figure 5.

As shown in Figure 4, when the arch height of a single bonding wire keeps increasing, $L_{tps\_lf}$ keeps increasing. The low-frequency self-inductance value of the bonding wire calculated by the analytical model was compared with the inductance value extracted by Ansys Q3D. There was an excellent correlation between the inductance values extracted by the two methods, with a maximum difference of approximately 1%.

**Figure 6:**
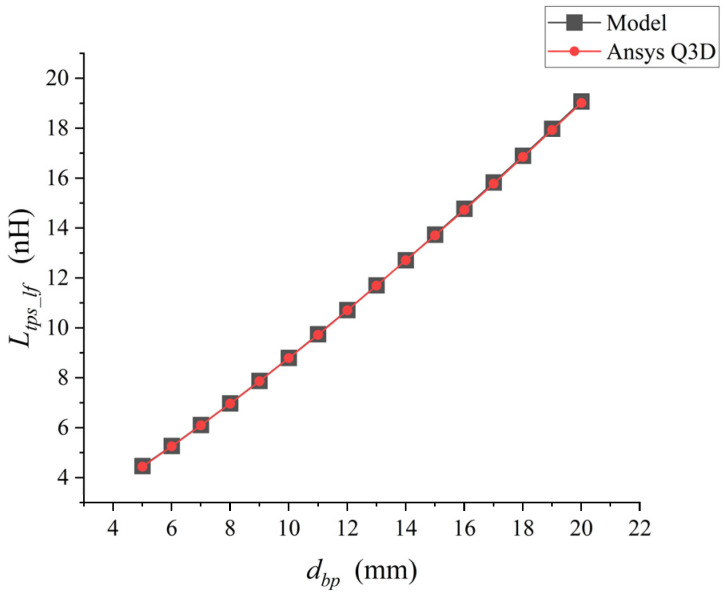
Point plot of the relationship between $d_{bp}$ and $L_{tps\_lf}$.

When $r_{bw}$ and $H_{max}$ remained unchanged and only $d_{bp}$ was changed, the analytical model was used for calculation in matlab and the simulation was conducted in Ansys Q3D, respectively. The comparison results are shown in Figure 6.

As shown in Figure 5, when the distance between the bonding points of a single bonding wire keeps increasing, its parasitic self-inductance $L_{tps\_lf}$ keeps increasing. The low-frequency self-inductance value of the bonding wire calculated by the analytical model was compared with the inductance value extracted by Ansys Q3D. There was an excellent correlation between the inductance values extracted by the two methods, with a maximum difference of approximately 1%.

### 3.2. Bonding Wires Mutual Inductance Verification

To verify the analysis model derived for the coupled bonding wire, the IGBT bonding wire configuration shown in Figure 3 was analyzed using Q3D and our analysis model.

**Figure 7:**
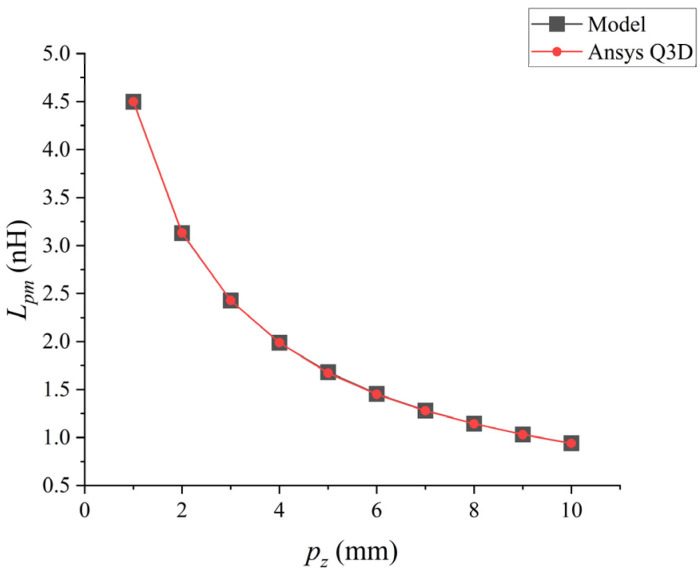
Point plot of the relationship between $p_{z}$ and $L_{pm}$.

Using the analytical model and Q3D we proposed, the mutual inductance values of the coupling bonding wire where only the *Z*-axis spacing $p_{z}$ changes were analyzed. The results are shown in Figure 7.

When $p_{z}$ expands from 1 mm to 10 mm, their parasitic mutual inductance decreases in an exponential form. The low-frequency mutual inductance value of the bonding wire calculated by the analytical model was compared with the inductance value extracted by Ansys Q3D. There was an excellent correlation between the inductance values extracted by the two methods, with a maximum difference of approximately 1%.

**Figure 8:**
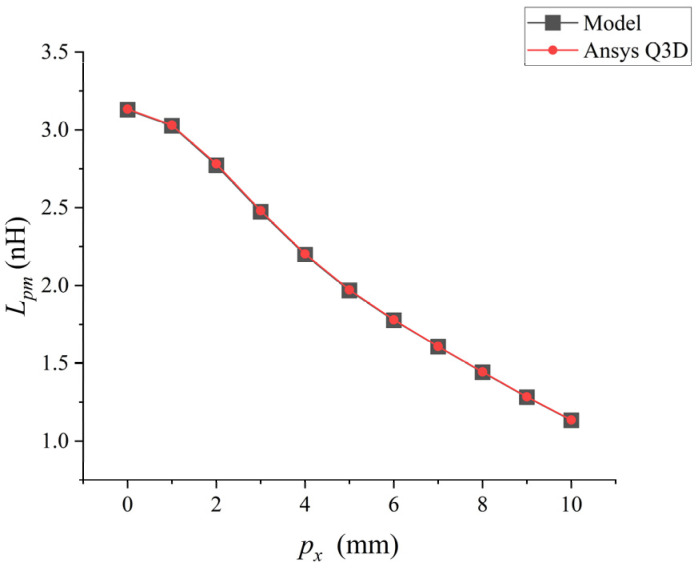
Point plot of the relationship between $p_{x}$ and $L_{pm}$.

Then, the mutual inductance values where only $p_{x}$ of the coupled bonding wire changes were considered for analysis, and the results are shown in Figure 8. At this point, the $p_{z}$ of the coupled bonding wire is set to 2 mm.

When $p_{x}$ increases from 0 mm to 10 mm, their parasitic mutual inductance shows a downward trend in Figure 8. The low-frequency mutual inductance value of the bonding wire calculated by the analytical model was compared with the inductance value extracted by Ansys Q3D. There was an excellent correlation between the inductance values extracted by the two methods, with a maximum difference of approximately 1%.

**Figure 9:**
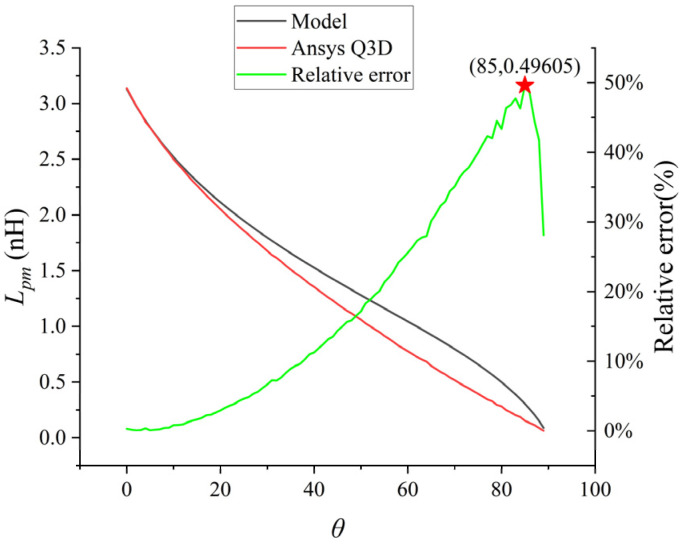
Point plot of the relationship between *θ* and $L_{pm}$.

Then, the partial mutual inductance values when only the deflection angle *θ* of the coupled bonding wire changed were analyzed, and the results are shown in Figure 9. At this point, the $p_{z}$ of the coupled bonding wire is set to 2 mm.

As shown in Figure 9, when *θ* gradually increases from 0 to 90 degree, their parasitic mutual inductance shows a downward trend. The low-frequency mutual inductance value of the bonding wire calculated by the analytical model was compared with the inductance value extracted by the numerical values of Ansys Q3D. At the beginning, the above two values were almost coincident, and then the difference gradually increased. The maximum difference was approximately 49.6%, and finally there was a close coincidence near 90 degree.

To enhance the calculation accuracy of the analytical model across the entire range of angles and make it more suitable for engineering design and rapid assessment, this study introduces an empirical correction factor k(θ) related to the deflection angle *θ* as shown in (19), and obtains a correction factor lookup table for key angles through calculation, as shown in Table 2. This table is spaced at 5° intervals and provides a convenient reference for practical applications.

(19) $$L_{\mathrm{corr}}(\theta )=k(\theta )\cdot L_{\mathrm{model}}(\theta ),$$

For the correction factor at any angle *θ*, it can be obtained through linear interpolation using the adjacent data in the table. After applying this correction scheme, the model accuracy has been significantly improved. Taking the error peak point *θ* = 85° as an example, by referring to the table, we obtain k(85°) = 0.5040. Substituting it into the calculation, we obtain $L_{\mathrm{corr}}(\theta )$ = 0.5040 × 0.30499 = 0.1537 nH, which is exactly the same as the Q3D simulation result (0.15370 nH). Statistical analysis shows that the maximum relative error after correction is controlled within 1%. Within the common actual deflection range of the bonding line (*θ* ≤ 45°), the error is even lower than 0.5%, fully meeting the precision requirements of engineering design.

### 3.3. The Relationship Between the Parasitic Inductance of Bonding Wires and Their Morphology

As mentioned earlier, the previous study on the parasitic inductance of bonding wires always treated the bonding wires as long straight wires for calculation. When the material, diameter and length of the long straight wires are the same, the calculated parasitic inductance results will be the same. Here, the bonding wires are controlled at the same length, and the influence of the shape of the bonding wires on the parasitic inductance of the bonding wires is studied.

**Figure 10:**
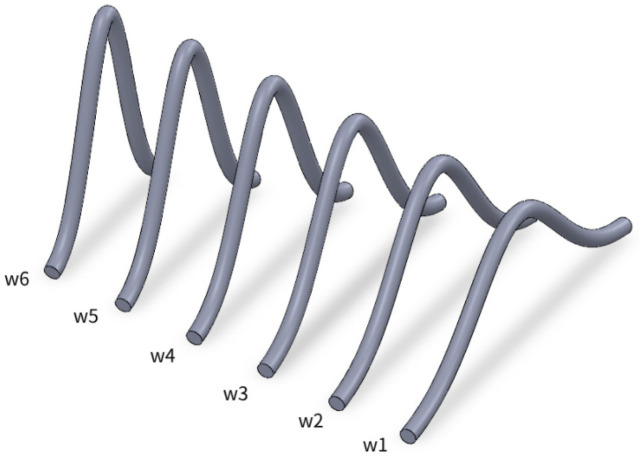
Bonding wires of the same $l_{bw}$ but with different $H_{max}$ and $d_{bp}$.

Firstly, taking the bonding wire manufactured with $r_{bw}\;$= 15 $mil,\;H_{max}\;$= 3 mm and $d_{bp}\;$= 10 mm as an example, the length of this bonding wire is calculated to be 11.8208 mm by (2), and its parasitic inductance is calculated to be 8.7959 nH by formula. When we keep the length of the bonding wire constant and change the ${Lh}_{max}$ and $d_{bp}$ of the bonding wire, we can produce five different forms of bonding wires as shown in Figure 10.

The parasitic inductance calculated by the analytical model and the simulation obtained by Ansys Q3D at a frequency of 10 kHz was compared to discuss the influence of the morphology of the bonding wire on the parasitic inductance of the bonding wire. The calculation results are shown in Table 3.

It can be inferred from Table 3 that when the length of the bonding wire is kept consistent, as the bonding wire keeps getting higher, the distance between the bonding points becomes smaller, and the parasitic self-inductance value of the bonding wire keeps decreasing, from the maximum 8.7959 nH to 6.1792 nH. The maximum difference between the calculation results of the analytical model and the simulation results of Ansys Q3D is approximately 1%.

**Figure 11:**
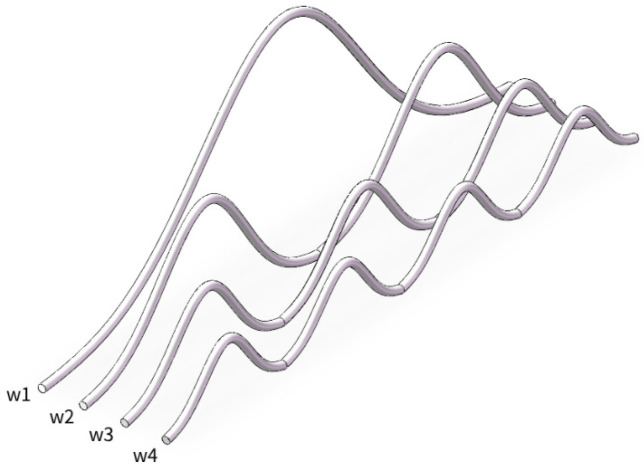
Bonding wires of the same $l_{bw}$ and $d_{bp}$ but with different $H_{max}$.

Taking the bonding wire manufactured with $r_{bw}$= 15 $mil,\;H_{max}$= 3 mm and $d_{bp}$= 10 mm as an example, the length of the bonding wire is calculated to be 37.3862 mm by formula (2), and its parasitic inductance is calculated to be 35.263 nH. When we change the total length of the bonding wire to remain unchanged, the shape of the changed bonding wire is shown in Figure 11.

Figure 11 shows that the long wire with only one arch will change its shape into a long wire with several arches. The $H_{max}$ of the bonding wire will decrease, but the length of the bonding wire remains unchanged. The parasitic self-induction of several forms of bonding wires was calculated using the analytical model and compared with the simulation results of Ansys Q3D at a frequency of 10 kHz. The calculation results are shown in Table 4.

### 3.4. Research on the Total Inductance of Bonded Wire Arrays

When there are only two bonding wires in the space connected in parallel and passing through the same direction of current, the total inductance is as shown in (20). When the two bonding wires are exactly the same, that is, $L_{1}$ = $L_{2}$ = *L*, the (20) simplified to (21). However, when the bonding wires pass through an opposite direction of current, the total inductance is as shown in (22).

(20) $$L_{total}=\frac{L_{1}L_{2}-M^{2}}{L_{1}+L_{2}+2M},$$

(21) $$L_{total}=\frac{L+M}{2},$$

(22) $$L_{total}=\frac{L-M}{2},$$

**Figure 12:**
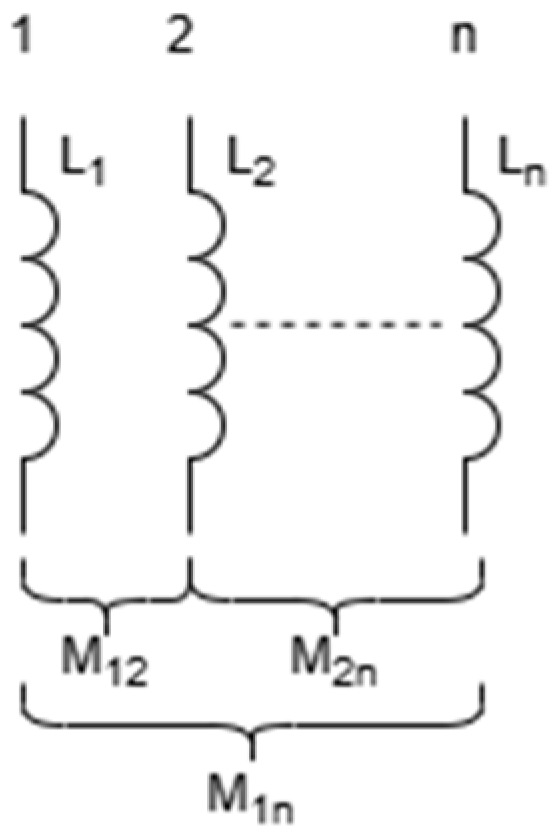
Schematic diagram of the parasitic inductance of the bonded wire array.

In the internal structure of IGBT modules, multiple bonding wires are usually connected in parallel to form a bonding wire array, enabling the IGBT chip and FRD (Fast Recovery Diode) chip to be connected to DBC (Direct Bonded Copper). To establish a computable mutual inductance model, this study made necessary simplifications to the excitation conditions of the parallel bonded wires. Here, the following assumptions are made: Each bonded wire is ideally symmetrical in terms of geometry, material, and welding points. Therefore, under high-frequency or static approximations, the amplitude of the current flowing through each bonded wire is equal. This current equalization assumption simplifies the complex distributed coupling system into a symmetric excitation problem, which is the standard method adopted by many classical parasitic parameter analytical models. Although in practical work, due to process deviations and uneven electromagnetic coupling, the current distribution will change dynamically [31], this model aims to provide a fast and intuitive performance evaluation benchmark for the initial layout design stage. In typical application scenarios where geometric symmetry is guaranteed, this simplified model has engineering reference value. The schematic diagram of the bonding wire array is shown in Figure 12.

As shown in (23), the parasitic inductance of the bonding wire array not only exists in the self-inductance *L_n_* of the bonding wires themselves, but also in the mutual inductance *M*_12_, *M*_13_… The calculation formula for *M_nn_*, the total inductance, is shown in (24).

(23) $$\left[\begin{array}{cccc} L_{1} & M_{12} & \ldots & M_{1n}\\ M_{21} & L_{2} & \ldots & M_{2n}\\ \vdots & \vdots & \ddots & \vdots \\ M_{n1} & M_{n2} & \ldots & L_{n} \end{array}\right],$$

and

(24) $$L_{total}=\frac{1}{n^{2}}(\sum_{i=1}^{n}L_{i}+\sum_{i=1}^{n}\sum_{\begin{array}{c} j=1\\ j\neq i \end{array}}^{n}M_{ij}),$$

**Figure 13:**
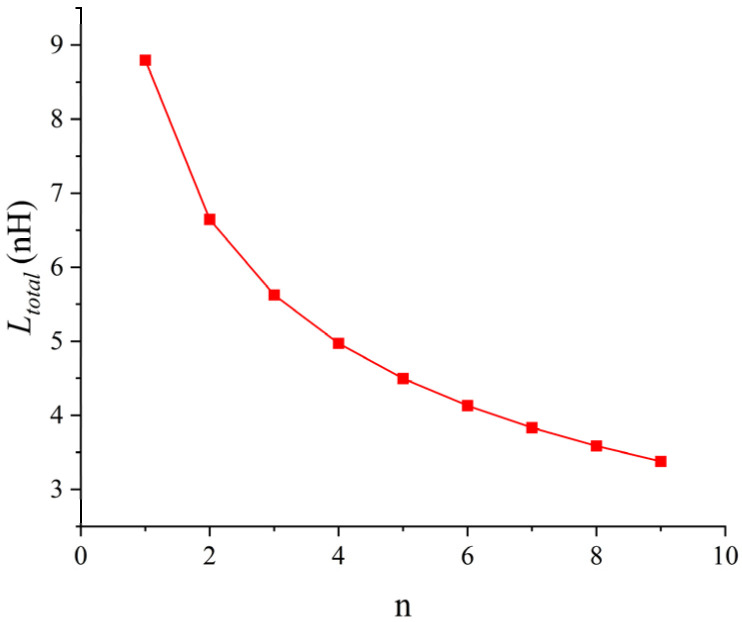
Point plot of the relationship between $L_{total}$ and the number of parallel connections *n*.

Here, taking the bonding wire fabricated with $r_{bw}\;$= 15 $mil,\;H_{max}\;$= 3 mm and $d_{bp}\;$= 10 mm as an example, when *n* bonding wires are connected in parallel and the $p_{z}$ between the bonding wires is 2 mm, the total parasitic inductance $L_{total}$ of the bonding wire array is shown in Figure 13.

As can be seen from Figure 13, as the number of parallel bonding wires *n* increases, the total parasitic inductance $L_{total}$ of the bonding wire array continuously decreases, but the extent of this reduction is also declining. When the number of parallel bonding wires exceeds 5, the extent of this reduction drops from the initial 24.4% to 9.5%.

## 4. Research on the Layout of Bonding Wires

This section studies the layout of bonding wires by using the analytical models for calculating self-inductance and mutual inductance of bonding wires and the research content on the morphology of bonding wires in the previous three sections.

### 4.1. Bonding Under Limited Width

When bonding wires on an IGBT chip, the area of the chip is limited, which means the number of bonding wires that can be bonded is also limited. When the number of bonding wires is fixed, the spacing between the bonding wires can be determined. From the previous research, the mutual inductance of the bonding wires decreases as the distance between the bonding wires increases. Based on the above points, the content of this summary is to study the relationship between the total parasitic inductance of the bonding wire array and the number and spacing of the bonding wires (the bonding width and current-carrying capacity of the IGBT chip are constant).

According to the literature [32], the current-carrying capacity of n bonding wires connected in parallel is $\sqrt{n\sqrt{n}}$ times that of a single bonding wire. To ensure the normal operation of an IGBT chip, a certain magnitude of current is required. Therefore, here, the number of bonding wires n needed for the current-carrying capacity of n bonding wires connected in parallel is set as the minimum required quantity. When the aluminum wire is bonded to the surface of a chip by the bonding machine, due to pressure, the bonding wire will undergo a certain deformation at the bonding point. According to the production standard, the maximum deformation is set to 1.5 times the diameter of the bonding wire here. Here, the bonding width of the chip is divided by 1.5 times the diameter of the bonding wire, and the resulting number of bonding wires is the maximum required quantity. However, the more bonding wires there are, the smaller the distance between them becomes, and the greater the actual processing difficulty. Therefore, the minimum required quantity plus 5 is taken as the maximum required quantity.

**Figure 14:**
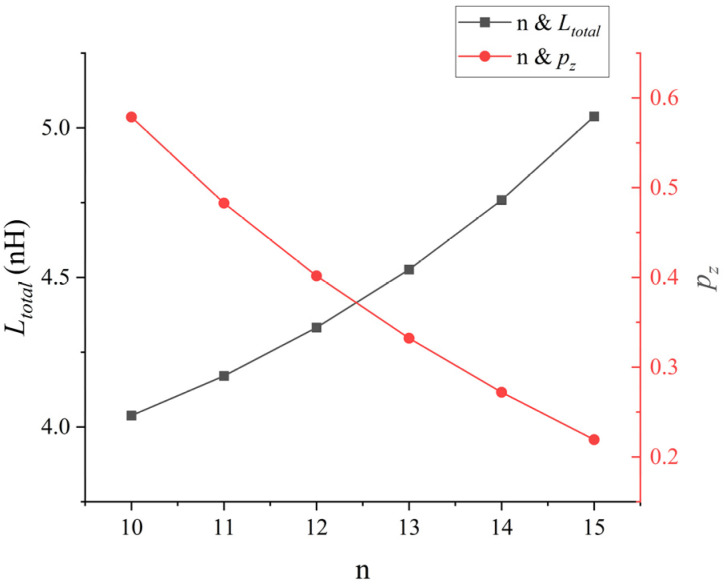
Point plot of the relationship between $L_{total}$, *n* and $p_{z}$.

In this section, aluminum wires with $r_{bw}\;$= 15 $mil,\;H_{max}\;$= 3 mm and $d_{bp}\;$= 10 mm are used as bonding wires. The current-carrying capacity of the IGBT chip is 120 A, and the chip width is 12.08 mm. The calculation was carried out on MATLAB (R2022b), and the calculation results are shown in Figure 14.

As shown in Figure 14, according to the calculation on the MATLAB, the minimum number of bonding bonds on the IGBT chip is 10, with $p_{z}$ = 0.58 mm, and $L_{total}$ = 4.0382 nH. In addition, he maximum value is 15, with $p_{z}$ = 0.22 mm and $L_{total}$ = 5.0381 nH. The total parasitic inductance has increased by approximately 25%, and the distance between the bond wires has decreased by approximately 50%. From these results, when the bonding width and current-carrying capacity of the IGBT chip are constant, as the number of bonding wires *n* increases, the bonding wire spacing $p_{z}$ continuously decreases. Thus, the production difficulty increases, the total parasitic inductance of the bonding wire array continuously increases, and its manufacturing cost performance also continuously decreases.

Therefore, when designing the bonding wire array in practice, blindly increasing the number of bonding wires may increase the total current-carrying capacity of the array. However, when the bonding width of the IGBT chip is fixed, it is still necessary to appropriately consider the spacing of the bonding wire array, the total parasitic inductance, and the processing difficulty.

### 4.2. Arrays of Bonding Wires with Different Layouts

**Figure 15:**
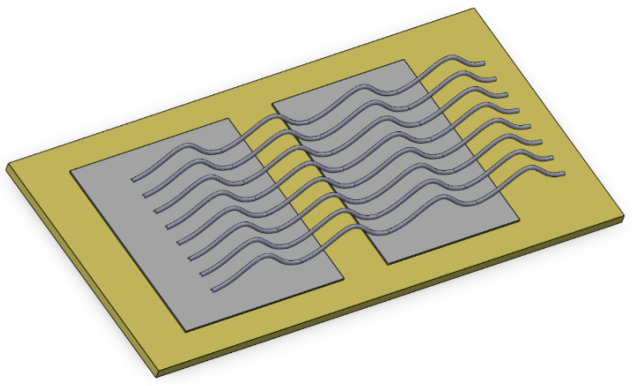
Typical interconnection structures of bonding wires with IGBT chips, FRD chips and DBC.

As shown in Figure 15, it is a typical IGBT chip and FRD chip interconnected with DBC bonding wires inside an IGBT module. In this section, the total parasitic inductance of this classical interconnection structure is studied.

**Figure 16:**
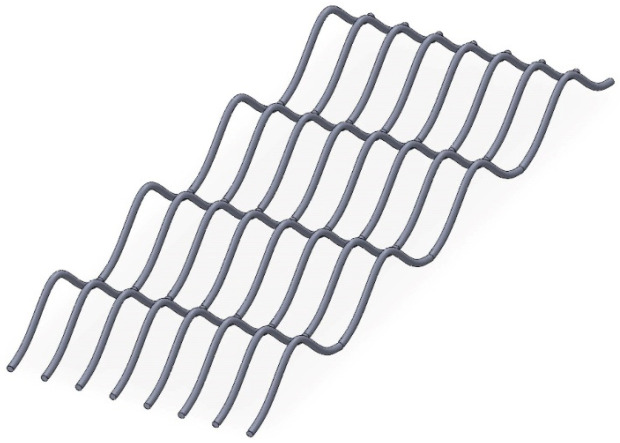
Typical bonding wires array structure.

**Figure 17:**
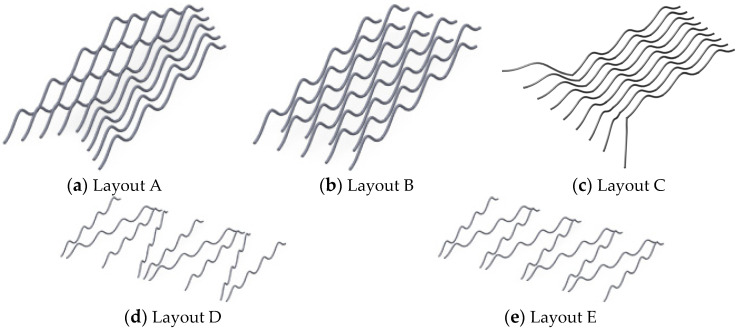
Arrays of bonding wires with different layout structures.

In Figure 16, there are nine bonding wires connected in parallel, with a spacing of 2 mm between each other. The arch height of each bonding wire is ${Lh}_{max}\;$= 3 mm and $d_{bp}\;$= 40 mm. The simulation was conducted using Ansys Q3D at a frequency of 10 kHz, and the simulation result was 18.1439 nH. Figure 17 lists five variant layouts.

For the bond wires layout A of Figure 17a, the even sequence bond wires differ by 5 mm from the odd sequence bond wires on the *X*-axis, while in the bond wire layout B of Figure 17b, each bond wire differs by 2 mm on the *X*-axis, presenting a v shape. The simulation was conducted using Ansys Q3D at a frequency of 10 kHz. The simulation results were 17.4207 nH and 17.7102 nH, respectively, which were reduced by approximately 4% and 2.4%, respectively, compared with the typical layout.

In Figure 17c, the differences in the bonding wires array layout C only occur at the final bonding point. From top to bottom, it gradually shifts from a +45 degree angle to a −45 degree angle. The simulation was conducted using Ansys Q3D at a frequency of 10 kHz. The simulation results were 17.8766 nH, which was approximately 1.5% less compared to the typical layout.

In the bond wires layouts D and E of Figure 17d,e, the deflection angle between the bond wires is 15 degrees. The difference between the two lies in the different arrangements, which result in parasitic inductances of 17.6967 nH and 11.6096 nH, respectively, reducing by approximately 2.4% and 36% compared to the typical layout.

## 5. Conclusions

In this paper, based on the consideration of factors such as the actual shape of the bonding wire, connection parameters, and spatial parameters, a new analysis model was derived and verified. These models can accurately and efficiently calculate the parasitic inductance value of the bonding wire. Using this new analysis model, the influence of the shape modeling of the bonding wire on the parasitic self-inductance was analyzed. On the basis of these studies, several new bonding wire arrays were listed and derived, and the influence of the parameters mentioned in the analysis model on the total parasitic inductance of the bonding wire array was verified. The analysis model proposed in this paper can reduce the impact of bonding parameters on the total parasitic inductance of the bonding wire during the pre-layout stage, providing certain assistance for the research and development of IGBT module packaging.

It should be noted that this research is mainly based on simulation verification, which is a common and necessary method during the stage of concept formulation and principle validation. Looking ahead, the reasonable and critical expansion of this work lies in integrating it into a comprehensive reliability collaborative design framework. The parasitic inductance values calculated by the model we proposed are important input data for subsequent electro-thermal coupling simulations. Our future research will focus on combining the existing inductance model with thermal and thermal-mechanical models to quantitatively analyze the heating caused by switch losses on the wire temperature, stress, and long-term reliability. This integrated approach, based on the foundation of the established electromagnetic analysis, will support an overall packaging design strategy that simultaneously optimizes electrical performance and reliability.

## Figures and Tables

**Table 1:** Comparison of bond wire morphology and calculation method in references.

Bonding Wire Morphology	Calculation Method	References
long straight wire	Grover Formula	[13,14]
straight transmission segments	Grover Formula	[15]
semicircles/half-circular loops	PEEC	[16,17]
ball bonding form	MoM	[18,19]
single-wire and double-wire electrical models	FDTD	[20]
the Gaussian distribution model	Magnetic flux integral	[21]

**Table 2:** Bonding wire mutual inductance model correction factor k(θ).

*θ* (°)	K (*θ*)	*θ* (°)	K (*θ*)	θ (°)	K (*θ*)
0	1.0030	30	0.9333	60	0.7442
5	0.9992	35	0.9108	65	0.6988
10	0.9918	40	0.8874	70	0.6493
15	0.9837	45	0.8561	75	0.6009
20	0.9707	50	0.8283	80	0.5664
25	0.9538	55	0.7865	85	0.5040

**Table 3:** Same $l_{bw}$ with different $H_{max}$ and $d_{bp}$.

Wire Number n	$d_{bp}$ [mm]	$H_{max}$ [mm]	$L_{tps\_lf}$ (Matlab) [nH]	$L_{tps\_lf}$ (Q3D) [nH]
1	10	3	8.7959	8.78484
2	9	3.6441	8.3026	8.28877
3	8	4.1566	7.8016	7.78433
4	7	4.5823	7.2876	7.26417
5	6	4.9420	6.7516	6.71648
6	5	5.2468	6.1792	6.13130

**Table 4:** Same $l_{bw}$ and $d_{bp}$ with different $H_{max}$.

Wire Number n	$l_{bw}$ [mm]	$H_{max}$ [mm]	$L_{tps\_lf}$ (Matlab) [nH]	$L_{tps\_lf}$ (Q3D) [nH]
1	37.3862	10	37.3862	35.2487
2	37.3862	5.8955	35.1178	35.1573
3	37.3862	3.9303	34.1683	34.1065
4	37.3862	2.9477	33.5456	33.4674

## Data Availability

The original contributions presented in this study are included in the article. Further inquiries can be directed to the corresponding author.
